# The Peroxymonocarbonate Anion HCO_4_^−^ as an Effective Oxidant in the Gas Phase: A Mass Spectrometric and Theoretical Study on the Reaction with SO_2_

**DOI:** 10.3390/molecules28010132

**Published:** 2022-12-23

**Authors:** Chiara Salvitti, Federico Pepi, Anna Troiani, Marzio Rosi, Giulia de Petris

**Affiliations:** 1 Dipartimento di Chimica e Tecnologie del Farmaco, “Sapienza” University of Rome, P.le Aldo Moro 5, 00185 Rome, Italy; 2 Dipartimento di Ingegneria Civile ed Ambientale, University of Perugia, Via Duranti 93, 06125 Perugia, Italy

**Keywords:** peroxymonocarbonate anion, sulphur dioxide, oxidation reactions, electrospray ionisation mass spectrometry, ion–molecule reactions, theoretical calculations

## Abstract

The peroxymonocarbonate anion, HCO_4_^−^, the covalent adduct between the carbon dioxide and hydrogen peroxide anion, effectively reacts with SO_2_ in the gas phase following three oxidative routes. Mass spectrometric and electronic structure calculations show that sulphur dioxide is oxidised through a common intermediate to the hydrogen sulphate anion, sulphur trioxide, and sulphur trioxide anion as primary products through formal HO_2_^−^, oxygen atom, and oxygen ion transfers. The hydrogen sulphite anion is also formed as a secondary product from the oxygen atom transfer path. The uncommon nucleophilic behaviour of HCO_4_^−^ is disclosed by the Lewis acidic properties of SO_2_, an amphiphilic molecule that forms intermediates with characteristic and diagnostic geometries with peroxymonocarbonate.

## 1. Introduction

The peroxymonocarbonate anion, HCO_4_^−^, was first isolated from alkali metal salts [1,2] and characterised by NMR, Raman spectroscopy [3], and X-ray crystallography [4] as the covalent adduct of the hydroperoxide anion HO_2_^−^ and CO_2_ (HOOCO_2_^−^). This anionic peracid, whose structure resembles that of analogous peroxo-species (e.g., peroxymonosulfate, peroxynitrate, and peroxyacetate) [5], is formed by the in situ reaction between hydrogen peroxide and bicarbonate at a near-neutral pH and in the absence of strong and corrosive acids [6]. Accordingly, bicarbonate-activated hydrogen peroxide (BAP) systems provide a source of HCO_4_^−^ ions, which are employed as a selective electrophilic oxidant for electron-rich compounds including alkenes [7], amines [8], organic sulphides [9,10,11], and thiols [12]. HCO_4_^−^ is also widely exploited in field-scale applications in environmental remediation processes [13,14] and water treatment [15]. No less important is its role in biological systems, where both hydrogen peroxide and bicarbonate are ubiquitous species in extracellular fluids [16,17]. Indeed, HCO_4_^−^ has been indicated as a reactive oxygen species (ROS) in the two-electron oxidation of sulphur-containing aminoacidic residues [11,12], with important implications in neurodegenerative diseases [18] and in the peroxidase activity of copper and zinc superoxide dismutase enzymes (Cu, Zn-SOD) [19]. In most of the above cases, the peroxymonocarbonate ion acts as an electrophilic oxidant [7,8,9,10,11,12,13], while only a few studies report its behaviour as a nucleophile [20,21,22,23], although it is believed that the strong oxidant HOO^−^, present in BAP systems, is the actual nucleophilic oxidant [14].

In contrast to the wealth of studies performed in solution, in the gas phase, to the best of our knowledge, only one study has reported the reactivity of an isobaric electrostatic ionic complex [HO_2_⸱⸱⸱CO_2_] ^−^ towards atmospheric constituents [24], suggesting the possible occurrence of a bound species, the peroxymonocarbonate ion. 

In the gas phase, the peroxymonocarbonate anion has been known since 1977, when it was observed in a flame ionisation detector [25,26] upon clustering reactions involving free oxygen radicals with CO_2_ or carbonate derivatives, and more recently in air plasma [27,28], either as a free species or as a negative core ion of large water clusters [29]. The lack of gas-phase reactivity studies concerning HCO_4_^−^ ions prompted us to investigate the reactions of this anionic peracid in a joint mass spectrometric and theoretical approach. Over the years, this approach has provided a wealth of knowledge on the intrinsic reactivity of isolated ions towards neutral species in a pristine environment, i.e., in the absence of the masking effects of solvents or counter-ions typically operating in bulk environments [30,31,32]. We therefore undertook an investigation of the oxidative behaviour of HCO_4_^−^ towards sulphur dioxide by ion trap mass spectrometry, exploiting an in-house-modified experimental set-up to perform ion–molecule reactions [33]. This experimental approach allows one to precisely ascertain the identity of the reactive (oxidant) species, as well as the relationship between the reagent (oxidant), intermediates and the products involved [30,31]. In the last few years, we have thoroughly investigated the reactivity of sulphur dioxide, one of the most produced commodity chemicals worldwide [34] and, at the same time, a harmful pollutant [35], with singly [36,37] and doubly charged [38,39,40] metal oxide anions and non-metal anionic oxides [41,42,43,44]. The very rich chemistry observed is the result of the amphoteric nature of SO_2_, whose sulphur atom can either behave as an electron acceptor or as an electron donor. The nucleophilic ability is due to the lone pair on sulphur in a high-lying σ-based HOMO, whereas the electrophilic properties are due to the low-lying π-symmetry LUMO of sulphur dioxide [45]. Interestingly, the donor–acceptor behaviour of SO_2_ is reflected in the diagnostic geometrical features of non-metal [46] and metal–SO_2_ complexes [45,47].

We found that SO_2_ was effectively oxidised by HCO_4_^−^ to hydrogen sulphate, sulphur trioxide, and SO_3_^−^, together with carbon dioxide, hydrogen carbonate, and the hydrogen carbonate radical. Hydrogen sulphite was also observed as a result of a secondary reaction promoted by HCO_3_^−^ [48]. Our study adds a new contribution to the gas-phase chemistry of both HCO_4_^−^ and sulphur dioxide, highlighting the nucleophilic oxidative properties of peroxymonocarbonate, which are disclosed thanks to the electrophilic character of sulphur dioxide. A brief description of the generation of the anionic peroxide HCO_4_^−^ by electrospray ionisation (ESI) in our experimental set-up is also given, whereas experiments on sulphur-containing molecules of atmospheric, environmental, and biological interest are ongoing in our laboratory. 

## 2. Results

### 2.1. Formation and Characterisation of Peroxymonocarbonate Ion (HCO_4_^−^) 

The peroxymonocarbonate ion (HCO_4_^−^) is typically obtained in solution through a substitution reaction between bicarbonate (HCO_3_^−^) and hydrogen peroxide (H_2_O_2_) [6,7,9], whereas, in the gas phase, different formation pathways that consist of the oxidation of the CO_2_ or CO_3_^−^ species have been proposed [25,26,27,28]. Accordingly, we generated the HCO_4_^−^ ion by two alternative methods and precisely through the electrospray ionisation (ESI) of (1) a solution of sodium percarbonate (Na_2_CO_3_·1.5 H_2_O_2_; avail. H_2_O_2_ 20–30%) or (2) pure water.

The first approach is advantageous as sodium percarbonate is known to be a stable and easy-to-handle, commercially available compound, providing at once proper amounts of HCO_3_^−^ and H_2_O_2_ in water. The peroxymonocarbonate ion was indeed formed in Na_2_CO_3_·1.5 H_2_O_2_ solution and gently transferred to the gas-phase environment for structural and reactivity investigations.

The second option is also convenient since, by injecting pure water into the ESI source, line saturation effects and the need for frequent cleaning operations are avoided. Furthermore, the application of in-source electric fields to assess the ionisation process lead to the formation of different oxidising agents [49] that react with O_2_ or CO_2_ typically found in the ambient air or dissolved in water in different forms (CO_2(aq)_, HCO_3_^−^). The ESI approach is also a gentler ionisation method than atmospheric pressure chemical ionisation (APCI), which permits the application of a negative corona discharge in room air to obtain the HCO_4_^−^ ion [27,28,29,50]. According to these studies, we also succeeded in generating the peroxymonocarbonate ion through the APCI of ambient air, but the electrospray ionisation of water proved to be the best approach to assess the stable ionic signal of the HCO_4_^−^ species, which is a prerequisite for kinetic measurements. Interestingly, to the best of our knowledge, the formation of the HCO_4_^−^ ion under these conditions has never been emphasised by other authors, since the studies analysing the ESI speciation of pure water mainly investigate the aggregation phenomena of H^+^(H_2_O)_n_ and OH^−^(H_2_O)_n_ cluster ions [51]. 

**Figure 1:**
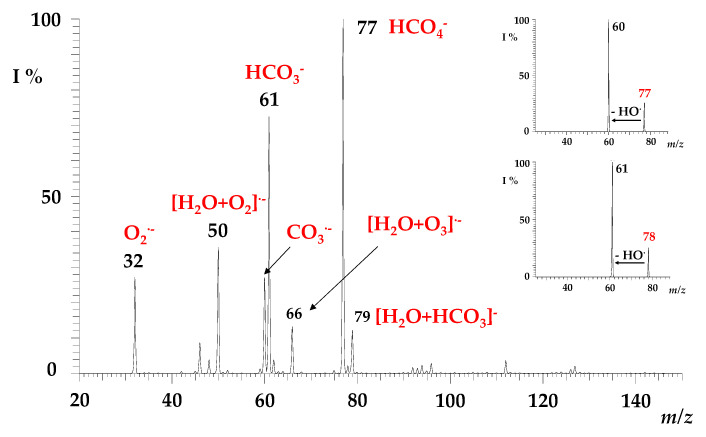
Mass spectrum for the electrospray of water acquired in the negative ion mode in the *m/z* range of 20–150. Inset: MS/MS of HCO_4_^−^ ions at *m/z* = 77 (upper) and H^13^CO_4_^−^ ions at *m/z* = 78 (lower). See text for details.

Focusing on the gas-phase formation pathways of the HCO_4_^−^ ion, two different reactions were proposed, namely the three-body association reactions of (1) CO_3_^−^ + OH^•^ + M and (2) HO_2_^−^ + CO_2_ + M [25,26,27,28,29]. In our experimental conditions, the ESI-(–) mass spectrum of pure water acquired in the low 20–150 mass range shows (Figure 1), among others, intense ionic signals at *m/z* 32 (O_2_^•−^), at *m/z* 50 (the hydrated cluster ion [O_2_·H_2_O]^•−^), and at *m/z* 60 corresponding to the CO_3_^−^ ion. The HO_2_^−^ species at *m/z* 33, commonly obtained through the discharge ionisation of H_2_O/O_2_ mixtures [52], is present only in a very low amount, as also already reported in the corona discharge ionisation systems by Ninomiya et al. [28], together with other ionic species also present in the ESI-(-) mass spectrum, but not involved in the formation of the HCO_4_^−^ ion.

To evaluate the possible formation pathway occurring under ESI conditions, we carried out two different experiments. In the first, we infused aliquots of water (previously decarbonated) enriched with Na_2_^13^CO_3_ or with NaH^13^CO_3_ as a source of ^13^CO_3_^−^ ion. As a result, the HCO_4_^−^ ion at *m/z* 77 always largely predominates over the labelled H^13^CO_4_^−^ species at *m/z* 78, even if the H^13^CO_3_^−^ ion represents the base peak, thus excluding the CO_3_^−^ + OH^⸱^ + M reaction as a possible HCO_4_^−^ formation pathway.

In the second experiment, we analysed an aliquot of water previously degassed to eliminate the dissolved CO_2_ and subsequently placed in a closed vessel saturated with ^13^CO_2_ (P ^13^CO_2_ = 700 torr). Although the occurrence of the ^13^CO_2(g)_ ⇄ ^13^CO_2(aq)_ ⇄ H^13^CO_3_^−^_(aq)_ equilibrium in water is unavoidable [53], a strong increase in the H^13^CO_4_^−^ ion compared to its ^12^C isotopologue was observed, highlighting the occurrence of the HO_2_^−^ + CO_2_ + M reaction in the ESI generation of the peroxymonocarbonate ion. According to this hypothesis, the neutral CO_2_, rather than CO_3_^−^ or HCO_3_^−^ ions, is supposed to be the oxidation target, as demonstrated by the absence of an increase in the HCO_4_^−^ ionic signal when water is enriched with bicarbonate or carbonate species, as previously described. 

Interestingly, our results parallel the evidence of the intermediacy of CO_2_ in bicarbonate/hydrogen peroxide solutions, where HCO_4_^−^ is formed through CO_2_ perhydration or base-catalysed perhydration [6,8,11]. 

Prior to investigating its gas-phase reactivity (see next paragraph), the HCO_4_^−^ species was mass-selected and characterised by collision-induced dissociation (CID) experiments. The HCO_4_^−^ ion at *m/z* 77 fragments by releasing a hydroxyl radical counterpart (OH^•^; 17 Da) [28], leading to the formation of a carbonate radical anion (CO_3_^•−^) at *m/z* 60 (Figure 1, upper inset), which accounts for the higher EA of CO_3_^•^ with respect to HO^•^ [54]. Likewise, the fragmentation of the ^13^C-isotopologue H^13^CO_4_^−^ at *m/z* 78 gives rise to the corresponding ^13^CO_3_^•−^ species at *m/z* 61 (Figure 1, lower inset).

### 2.2. Reactivity of Peroxymonocarbonate Ion (HCO_4_^−^) towards Sulphur Dioxide (SO_2_)

#### 2.2.1. Mass Spectrometric Results

**Figure 2:**
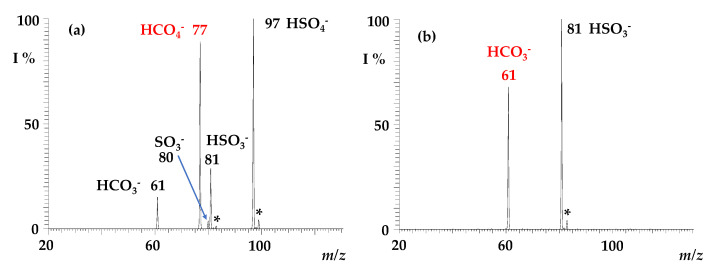
ITMS spectrum showing (**a**) the ion–molecule reaction of isolated HCO_4_^−^ ions (*m/z* 77) with SO_2_, reaction time = 200 ms, P SO_2_ = 1.3 × 10^−7^ Torr. Observed products: HSO_4_^−^ at *m/z* 97, HSO_3_^−^ at *m/z* 81, HCO_3_^−^ at *m/z* 61; the signals denoted with * correspond to H^34^SO_3_^−^ (*m/z* = 83) and to H^34^SO_4_^−^ (*m/z* = 99); (**b**) the ion–molecule reaction of HCO_3_^−^ ions (*m/z* 61) isolated from the sequence 77 → 61 with SO_2_ (reaction time = 200 ms, P SO_2_ = 1.3 × 10^−7^ Torr), showing that HSO_3_^−^ at *m/z* 81 is formed from a consecutive reaction. The signal denoted with * at *m/z* 83 corresponds to H^34^SO_3_^−^.

Regardless of the preparation method (if formed by electrospray ionisation of a percarbonate solution, of water, or of a saturated solution of ^13^CO_2_), the peroxymonocarbonate HCO_4_^−^ and its heavy ^13^C-form predictably show superimposable reactivity towards sulphur dioxide. They were mass-selected and exposed to SO_2_ in the ion trap cell to monitor the formation of ionic products as a function of the activation time. A typical product ion spectrum is reported in Figure 2, showing the formation of ionic signals at *m/z* 97, 81, 80, and 61, respectively, attributed to HSO_4_^−^, HSO_3_^−^, SO_3_^−^, and HCO_3_^−^. Ions at *m/z* 97 and 81 clearly contain one sulphur atom, as witnessed by the characteristic signature of the ^34^S peak at [M + 2] (H^34^SO_4_^−^ *m/z* = 99; H^34^SO_3_^−^ *m/z* = 83), though not easily distinguishable for the low-intensity SO_3_^−^ peak at *m/z* = 80. 

**Figure 3:**
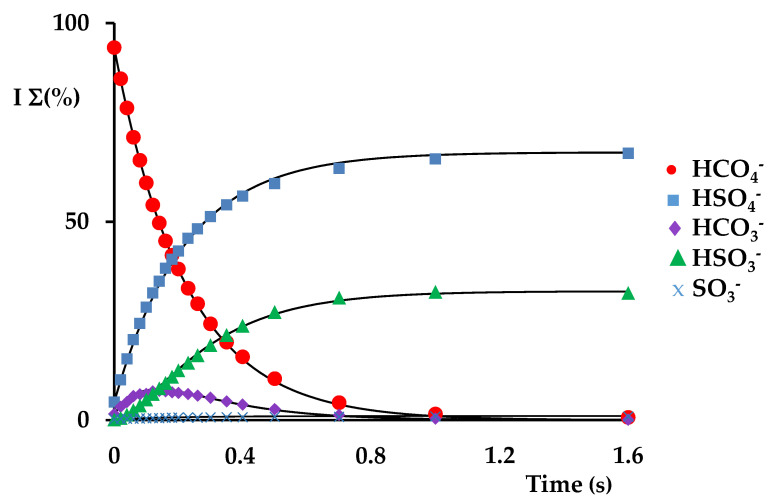
Kinetic plots and best fit lines of the reaction of isolated HCO_4_^−^ ions (*m/z* 77) (P SO_2_ = 1.97 × 10^−7^ Torr): ● HCO_4_^−^ (R^2^ = 0.9997), ■ HSO_4_^−^ (R^2^ = 0.9989); ▲ HSO_3_^−^ (R^2^ = 0.9946); ♦ HCO_3_^−^ (R^2^ = 0.9941); x SO_3_^−^ (R^2^ = 0.9764).

The kinetic profile of the reaction (Figure 3) shows that products are formed through parallel and consecutive reactions, and that the precursor ion conversion is fast and efficient, with a decay constant k*_dec_* at 298 K of 7.2 × 10^−10^ cm^3^ molecule^−1^ s^−1^ and efficiency (k/k*_coll_*) of 54.8% (Table 1). Three parallel reaction pathways can be identified in the following:

HCO_4_^−^ + SO_2_ → HSO_4_^−^ + CO_2_ (1)

HCO_4_^−^ + SO_2_ → HCO_3_^−^ + SO_3_ (2)

HCO_4_^−^ + SO_2_ → HCO_3_ + SO_3_^−^ (3)

The main reaction channel 1 consists of a formal SO_2_/CO_2_ switching, leading to the formation of the bisulphate HSO_4_^−^ product ion at *m/z* 97, accounting for a branching ratio of 64.5% (k_1_ = 4.6 × 10^−10^ cm^3^ molecule^−1^ s^−1^) and efficiency of 35.1% (Table 1). From another point of view, this reaction can also be considered as a formal hydroperoxide anion HOO^−^ transfer. Pathway 2 gives rise to the HCO_3_^−^ ion at *m/z* 61 with the release of SO_3_ as the neutral counterpart through an oxygen atom transfer (OAT) reaction to SO_2_ occurring with a rate constant of k_2_ = 2.5 × 10^−10^ cm^3^ molecule^−1^ s^−1^ (eff. = 19.1%) and a branching ratio of 34.8%. Finally, the very minor path 3, accounting for less than 1% of the products, leads to SO_3_^−^ at *m/z* 80, with a k_3_ = 5.0 × 10^−12^ cm^3^ molecule^−1^ s^−1^ (reaction 3, eff. = 0.4%) through oxygen ion transfer (OIT). The preferential formation of products from reaction 2 with respect to those of reaction 3 accounts for the considerably higher electron affinity of the bicarbonate radical (EA HCO_3_ = 3.68 eV) [55] with respect to SO_3_ (EA SO_3_ = 1.90 eV) [54].

The HCO_3_^−^ ion formed by reaction 2 is known to be reactive towards SO_2_ to form HSO_3_^−^ [48], and indeed it is rapidly depleted in a consecutive reaction forming the bisulphite anion at *m/z* 81 and carbon dioxide (Equation (4)), as illustrated in Figure 2b.

HCO_3_^−^ + SO_2_ → HSO_3_^−^ + CO_2_ (4)

This process occurs at a collision rate, with a measured rate constant k_4_ of 1.40 × 10^−9^ cm^3^ molecule^−1^ s^−1^ (Table 1), in good agreement with previous data [48]. Spectra relative to the reaction of the H^13^CO_4_^−^ ion are reported in Figure S1 (Supplementary Materials).

The reaction of HCO_4_^−^ + SO_2_ → HSO_4_^−^ + CO_2_ described in Equation (1) is an irreversible process, since no displacement of SO_2_ by CO_2_ occurred when the HSO_4_^−^ ion was reacted with CO_2_, not even with the high pressure of carbon dioxide or extension of the activation time up to the maximum value of 10 s. Similar irreversible switching reactions were also observed in the gas phase by reacting carbonate cluster ions with SO_2_ and resulting in the complete conversion of carbonate into sulphite ions [43].

#### 2.2.2. Theoretical Calculations

**Figure 4:**
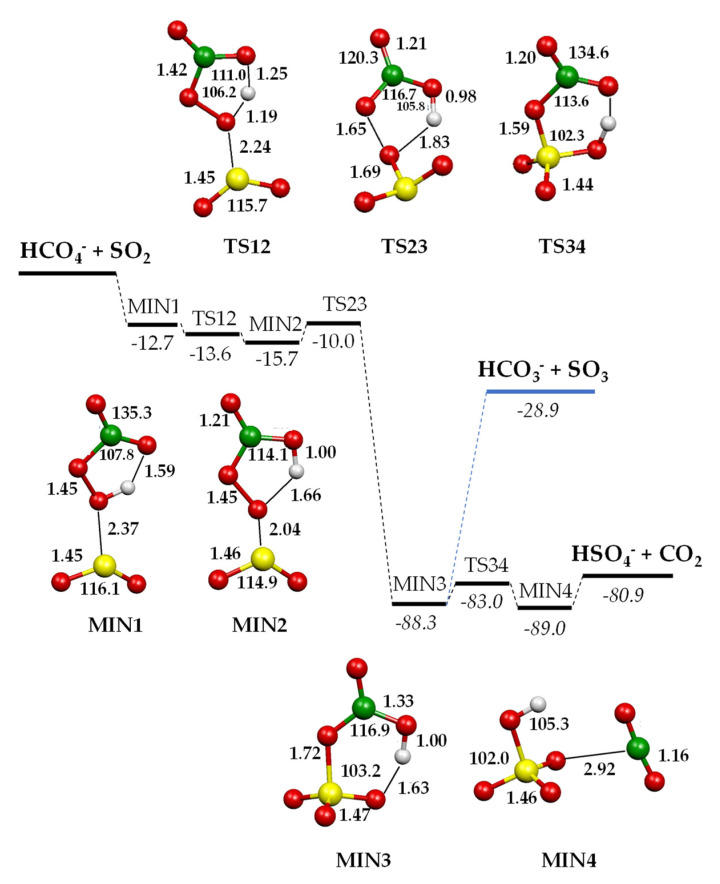
Schematic energy diagram of the reaction of HCO_4_^−^ ions with SO_2_. Geometries of the minima localised on the [HCO_4_···SO_2_]^−^ potential energy surface optimised at the B3LYP/aug-cc-pV(T+d)Z level of theory. Bond lengths in Å, angles in degrees, Δ*H*° values in kcal mol^−1^ computed at the CCSD(T)/aug-cc-pV(T+d)Z level of theory.

**Figure 5:**
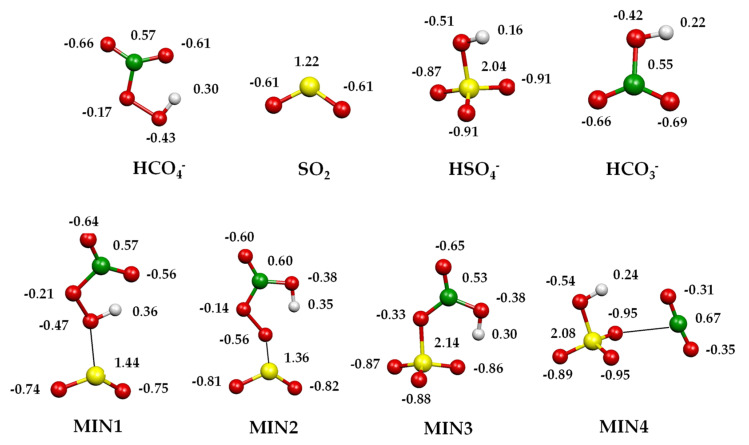
Charge density distributions of the minima and transition states localised on [HCO_4_···SO_2_]^−^ potential energy surfaces computed at the CCSD(T)/aug-cc-pV(T+d)Z level of theory.

The oxidation routes of SO_2_ promoted by HCO_4_^−^ were investigated using density functional calculations. Optimised geometries of relevant stationary points and transition states on the B3LYP/aug-cc-pV(T+d)Z potential energy surface for the processes are reported in Figure 4, while charge density distributions of relevant minima are shown in Figure 5. Complete geometrical parameters of minima and saddle points are shown in Figure S2 (Supplementary Materials). Enthalpy changes (kcal mol^−1^) at 298.15 K computed at the B3LYP/aug-cc-pV(T+d)Z and CCSD(T)/aug-cc-pV(T+d)Z levels of theory are detailed in Table 2. In the following, only the CCSD(T) values will be considered.

HCO_4_^−^ features a five-membered-ring intramolecular hydrogen bond between the carbonyl oxygen and the hydrogen atom bound to the peroxidic oxygen [28]. The initial attack of the peroxymonocarbonate anion involves this oxygen atom interacting with the incoming SO_2_ and leads to an encounter complex (MIN1) stabilised by a binding energy of 12.7 kcal mol^−1^. The two moieties, at a distance of 2.37 Å, almost conserve their original geometric features, with SO_2_ binding to the peroxyl oxygen in a bent coordination mode: the angle between the peroxyl O–S bond vector and the SO_2_ plane is around 100°, with the dihedral OSOO angle equal to 102.1°. With a negative small activation barrier, MIN1 converts to a slightly more stable MIN2, in which the proton is now bound to the carbonyl oxygen. The saddle point TS12 is highly stabilised by the sharing of the proton between the two oxygen atoms. In MIN2, the peroxidic O–S bond shortens to 2.04 Å, developing a small negative charge on SO_2_ (−0.27 e^−^) and still binding with a bent geometry (angle between peroxyl O–S bond vector and the SO_2_ plane is 100°, with the OSOO angle equal to 100.1°). In MIN2, an incipient SO_3_ moiety can be recognised and the charge density distribution on the two entities, SO_3_ and HCO_3_, is _−_0.83 e^−^ on the former and −0.17 e^−^ on the latter, therefore defining a sulphur trioxide anion and a bicarbonate radical. Proceeding along the reaction coordinate, MIN2 easily converts into MIN3, overcoming a barrier of only 5.7 kcal mol^−1^. In TS23, the easy formation of an S–O bond and the breaking of the O–O bond lead to a pyramidal SO_3_ and an HCO_3_ moiety. From this saddle point, a second oxygen atom is transferred from the carbonate to the sulphur trioxide moiety, forming an incipient sulphate in MIN3. This minimum is highly stabilised by 88.3 kcal mol^−1^ (Table 2) with respect to the entrance asymptote, thanks to the formation of a covalent S–O bond in the SO_3_ moiety and to the strong interaction of the sulphur atom with the oxygen of the bicarbonate, preluding at the formation of a sulphate species. MIN3 is further stabilised by an intramolecular hydrogen bond, forming a six-membered ring. The charge density distribution of this stationary point is almost equally distributed between the SO_3_ (−0.47 e^−^) and HCO_3_ (−0.53 e^−^) groups; nonetheless, in MIN3, one can also consider a (distorted) tetrahedral SO_4_ and a bent HCO_2_ moiety. Therefore, two competing paths leading to the final products can be envisaged from this point. The first is the dissociation of MIN3 into HCO_3_^−^ + SO_3_ or into HCO_3_ + SO_3_^−^, which is driven by the difference in AE between HCO_3_ and SO_3_. Accordingly, the exit path into HCO_3_^−^ + SO_3_ experimentally observed (reaction 2) largely prevails over the product couple HCO_3_ + SO_3_^−^, accounting for the much higher electron affinity of the bicarbonate radical (EA HCO_3_^•^ = 3.68 eV) [55] with respect to SO_3_ (EA SO_3_ = 1.90 eV [54], 2.22 eV [56]). The difference in the EA values of SO_3_ is due to the uncertainty attached to the heat of formation of SO_3_^−^ [54,56]. Accordingly, the enthalpy of reaction 3 is slightly positive (see also Table 2), which accounts for the low branching observed. A quite high dissociation energy of MIN3 (59.4 kcal mol^−1^, Table 2) is required both to break the hydrogen bond and the S–O bond, and to change the SO_3_ geometry from pyramidal to trigonal planar, to eventually release sulphur trioxide and the bicarbonate anion. This dissociation energy is, nevertheless, provided by the exothermicity of the process, calculated to be 28.9 kcal mol^−1^ (Table 2). Alternatively, MIN3 can isomerise with only 5.3 kcal mol^−1^ to the isoenergetic MIN4, a loose ion-neutral complex between the hydrogen sulphate anion and CO_2_, from which only 8.1 kcal mol^−1^ is required to release the products. The products HSO_4_^−^/CO_2_ are formed in a very exothermic process, calculated to be 80.9 kcal mol^−1^ (Table 2). Regarding the competition between the two product couples, HSO_4_^−^/CO_2_ and HCO_3_^−^/SO_3_, the experiments revealed a branching ratio of 3:1. Accordingly, the most stable HSO_4_^−^/CO_2_ products are also formed through the faster path, occurring through a reaction coordinate developing well under the entrance asymptote, whereas HCO_3_^−^/SO_3_, although forming from direct dissociation, requires a quite large amount of energy to attain the geometry of the products.

## 3. Discussion

Sulphur dioxide is effectively oxidised in the gas phase by the peroxymonocarbonate ion to hydrogen sulphate, sulphur trioxide, and, in a very minor amount, to SO_3_^−^. The reactions proceed through formal HOO^−^, oxygen atom, and oxygen ion transfers, respectively. In the absence of solvent molecules, the reaction is found to proceed along a PES initially characterised by the formation of two encounter complexes (MIN1 and MIN2, Figure 4), in which the two reactants, SO_2_ and HCO_4_^−^, maintain their geometric features and charge distribution. Nevertheless, the geometry of these adducts can be taken as a diagnostic of the bonding mode of sulphur dioxide, i.e., whether SO_2_ behaves as a donor or acceptor molecule. In fact, in analogy with known complexes, metallic [46,47] or non-metallic [47], when SO_2_ acts as a Lewis acid, it forms complexes with bent geometries, while linear geometries characterise SO_2_, which behaves as a Lewis base.

In agreement, the coordination of HCO_4_^−^ to SO_2_ in MIN1 and MIN2 results in a bent geometry of the complexes (see Figure S3), the angle between the O-S bond vector and the plane of SO_2_ moiety being around 100° in both structures, thereby qualifying SO_2_ as a Lewis acid, accepting the lone pair of the oxygen atom in the OH group through its lowest unoccupied molecular orbital, and the interaction as HCO_4_^−^ → SO_2_. The binding energies of the two minima, 12.7 and 15.7 kcal mol^−1^ (Figure 4, Table 2), are also in line with those calculated for complexes with pure donors, such as ammonia or methyl-substituted *N*-heterocyclic carbene, ranging from 10 kcal mol^−1^ (NH_3_∙SO_2_) to 18 kcal mol^−1^ (NHC∙SO_2_) [47]. Therefore, the Lewis acidic character of SO_2_ discloses the nucleophilic nature of HCO_4_^−^, which has rarely been observed in solution studies [20,21,22] when not questioned [14].

In solution, the generally accepted mechanism of the oxidation promoted by peroxymonocarbonate follows that of electrophilic peroxides [5]. The reaction occurs via a solvent-aided oxygen transfer mechanism, which involves a nucleophilic attack by the substrate, Nu, at the electrophilic oxygen of HCO_4_^−^ with the formation of the oxidised substrate, NuO, and the bicarbonate ion. An example is given by the electrophilic oxidation of sulphides, substrates with clear nucleophilic properties, which selectively leads to sulfoxides [9]. Secondary oxidation to sulfones is due to O_2_^•−^, generated upon the decomposition of HCO_4_^−^, or to H_2_O_2_, used to activate NaHCO_3_, rather than the peroxymonocarbonate itself [14]. Much uncertainty regarding the nucleophilic behaviour of HCO_4_^−^ still exists, also regarding the precise nature of the oxidant. Accordingly, the oxidative degradation of organophosphorus compounds, such as Paraoxon, in the NaHCO_3_-activated H_2_O_2_ solutions appears to be attributable to HO_2_^−^ or HO^−^ and not to HCO_4_^−^ [14]. This problem is easily circumvented in the gas phase, where it is possible to select a precise oxidant, i.e., HCO_4_^−^, and study its reactivity towards a selected substrate, i.e., SO_2_.

The actual oxidation step of the reaction occurs only in MIN3, in which one peroxidic oxygen atom has been fully transferred to sulphur dioxide, and the second one is still shared by the sulphur and carbon atoms. Accordingly, MIN3 can directly dissociate into HCO_3_^−^ + SO_3_ or into HCO_3_ + SO_3_^−^, with a branching that reflects the thermodynamic stability of the products, or it can evolve, through a small barrier, to a loose ion-neutral complex, eventually leading to the separate products HSO_4_^−^ + CO_2_. Hence, the driving force of the major, highly exothermic, oxidation process is the liberation of the stable carbon dioxide molecule. This circumstance can explain the different reactivity observed in the reaction of the strong nucleophile hydroperoxide, HOO^−^, which reacts with SO_2_ at collision rate, forming SO_3_^−^ and only a very small quantity of an adduct of formula HOOSO_2_^−^ and of unspecified structure [57].

Finally, the oxidation pathways described here show different characteristics compared to the SO_2_ oxidation promoted by other anionic systems, such as oxyhalogenated ions (XO_n_^−^ X = Cl, Br, I; n = 1,2) [42] or transition metal oxide anions (MO_2_^−^, M = Co, Ni, Cu, Zn; CrO_4_^−^) [37], whose common feature is that oxidation already occurs within the first highly exothermic intermediate, leading to free or bound SO_3_^0/−^. The same oxygen transfer process is not observed in the case of HCO_4_^−^, in which an intramolecular hydrogen bond engages the carbonyl oxygen in a stable 5-term cycle directing the reactivity towards the peroxidic OH that binds to SO_2_. Oxidation is only observed when the peroxidic oxygen is no longer bound to the hydrogen atom and can be eventually transferred to SO_2_.

## 4. Materials and Methods

### 4.1. Materials

The chemicals employed in this work were commercialised by Merck and used as received from the vendor. The stated purities are as follows: sodium percarbonate salt (Na_2_CO_3_·1.5 H_2_O_2_; avail. H_2_O_2_ 20–30%), Na_2_^13^CO_3_ (99 atom% ^13^C), NaH^13^CO_3_ (98 atom% ^13^C), ^13^CO_2_ (99 atom% ^13^C), and SO_2_ (99.9%). All the solvents (H_2_O HPLC grade, CH_3_CN HPLC grade) were purchased from Carlo Erba Reagents S.r.l. and not further purified.

### 4.2. Mass Spectrometry

Mass spectrometric investigations were performed by using an LTQ-XL linear ion trap mass spectrometer (ThermoFisher Scientific, Waltham, MA, USA) equipped with an electrospray ionisation (ESI) source. This instrument was previously adapted to realise ion–molecule reaction (IMR) experiments [33,36,37,38,39,40,41,42,43,44]. Accordingly, the gaseous neutral reactant can be introduced into the ion trap through a deactivated fused silica capillary entering the vacuum chamber from a 6.25 mm hole placed in the backside of the mass spectrometer. A Granville-Phillips Series 370 Stabil Ion Vacuum Gauge was used to measure the pressure of the neutral gas, which was kept constant by a metering valve. Owing to the position of the Pirani gauge compared to the ion trap, the actual gas pressure was obtained after reading calibrations [58]. The peroxymonocarbonate ion (HCO_4_^−^) was formed in solution by dissolving sodium percarbonate (Na_2_CO_3_·1.5 H_2_O_2_) in H_2_O/CH_3_CN (1:1, *v/v*) to a millimolar concentration, injected into the ESI source of the instrument at a flow rate of 5 μL min^−1^. Alternatively, the HCO_4_^−^ ion was obtained in the gas phase by directly subjecting pure water to the electrospray ionisation process. Nitrogen was used as a sheath and auxiliary gas at a flow rate of 11 and 2 arbitrary units (a. u.), respectively, a. u. ~0.37 L min^−1^. Other source parameters were set as follows: spray voltage 3.2 kV, capillary temperature 275 °C, tube lens 15 V, and capillary voltage 10 V. Once optimised, the HCO_4_^−^ reactant ion was isolated into the ion trap and exposed to gaseous sulphur dioxide (SO_2_). Typical SO_2_ pressures ranged between 1.1 × 10^−7^ Torr and 6.5 × 10^−7^ Torr, with uncertainty of ±30%. During the kinetic experiments, the normalised collision energy was set to zero, whereas the activation Q value was tuned to optimise the stable trapping fields for each ion. The ionic signals of the HCO_4_^−^ reactant and reaction products were monitored over time as a function of the SO_2_ concentration, and an average of 10 scans was acquired. Xcalibur 2.0.6 software was used to record and process all the mass spectra. Other selected neutrals, such as C_2_H_4_, ^13^CO_2_, C^18^O_2_, CO, and O_2_, proved unreactive with HCO_4_^−^. The reaction of HCO_4_^−^ ion with gaseous SO_2_ can be assimilated to a pseudo-first-order process due to the excess of neutral reactant compared to the precursor ion into the ion trap. Nonlinear least squares regression was performed by using the DynaFit4 software package [59], which simultaneously fit reactant and product concentrations vs. time. The reaction mechanism was verified by fitting the experimental results of the kinetic analysis with the mathematical model proposed for the postulated reaction, which simulated the time progress of the reaction based on the unimolecular rate constants obtained from the software calculations. The pseudo-first-order constants (s^−1^) were divided into the concentrations of neutral reagent gas to derive the bimolecular rate constants k (cm^3^ molecule^−1^ s^−1^), whereas the branching ratios of the different reaction channels were obtained from the formation constants of the direct products. According to the average dipole orientation (ADO) theory [60], the efficiency of the process was calculated as the ratio of the bimolecular rate constant k to the collision rate constant (k*_coll_*). Approximately 15 independent measurements were performed on different days over a sixfold neutral pressure range to guarantee the linearity of the k values. The standard deviation in the absolute rate constant was typically <10%, although a conservative error of 30% was given owing to the uncertainties affecting the measurement of the neutral pressure. The ionic reactant and products were also characterised by collision-induced dissociation (CID) experiments performed by furnishing energy to mass-selected ions in the presence of helium background gas (P He ca. 3 × 10^−3^ Torr). Normalised collision energies were tuned in a 20–40% range as a function of the isolated species and applied with a standard activation time of 30 ms. Ions were mass-selected with a window of 1 *m/z* and a Q value optimised to ensure stable trapping fields for all the ionic species under investigation.

The ^13^C-labelled experiments were performed by preparing and mass-analysing millimolar solutions of Na_2_^13^CO_3_ or NaH^13^CO_3_ salt. Analogous studies were also carried out on 1.5 mL of ultrasound-degassed water placed in a double bottle-neck flask that was filled with ^13^CO_2_ until a pressure of 700 torr.

### 4.3. Computational Details

The potential energy surface of the system [HCO_4_^−^···SO_2_] was investigated localising the lowest stationary points at the B3LYP [61,62] level of theory in conjunction with the correlation consistent valence polarised set aug-cc-pVTZ [63,64,65], augmented with a tight *d* function with exponent 2.457 for the sulphur atom [66], to correct for the core polarisation effects [67]. This basis set will be denoted aug-cc-pV(T+d)Z. At the same level of theory, we computed the harmonic vibrational frequencies in order to check the nature of the stationary points, i.e., minimum if all the frequencies are real, saddle point if there is one, and only one for the imaginary frequency. The energy of all the stationary points was computed at the higher level of calculation CCSD(T) [68,69,70] using the same basis set, aug-cc-pV(T+d)Z. Both the B3LYP and the CCSD(T) energies were corrected to 298.15 K by adding the zero point energy and the thermal corrections computed using the scaled harmonic vibrational frequencies evaluated at the B3LYP/aug-cc-pV(T+d)Z level. All calculations were performed using Gaussian 09 [71], while the analysis of the vibrational frequencies was performed using Molekel [72,73].

## 5. Conclusions

Sulphur dioxide is efficiently oxidised in the gas phase by the peroxymonocarbonate ion, HCO_4_^−^, through the formal transfer of HO_2_^−^, oxygen atom, and oxygen ion, to the hydrogen sulphate anion, sulphur trioxide, and sulphur trioxide anion, respectively. Secondary reactions due to the formation of the hydrogen carbonate anion lead to the formation of hydrogen sulphite. The three oxidative pathways have been characterised using a joint approach of mass spectrometry and theoretical calculations. Contrary to the common behaviour observed in solution, in the gas phase, the peroxymonocarbonate exhibits nucleophilic properties, which are unravelled by the Lewis acid character of sulphur dioxide.

## Figures and Tables

**Table 1:** Rate constants (cm^3^ s^−1^ molecule^−1^), efficiencies (%), and branching ratios (% Σ) relative to the reaction of HCO_4_^−^ + SO_2_. See text for rate constant numbering, k_n_.

	Rate Const. (×10^−10^) ^a^	Eff.% ^b^	Branching Ratio%
k*_dec_*	7.2	54.8	
k_1_	4.6	35.1	64.5
k_2_	2.5	19.1	34.8
k_3_	0.050	0.4	0.70
k_4_	14.0 ^c^	>100	

^a^ ±30%; ^b^ k/k*_coll_*; ^c^ see ref. [48].

**Table 2:** Enthalpy changes (kcal mol^−1^, 298.15 K) computed at the B3LYP/aug-cc-pV(T+d)Z and CCSD(T)/aug-cc-pV(T+d)Z levels of theory for selected reaction of HCO_4_^−^ + SO_2_.

	Δ*H*°_298.15_		Barrier Height	
	B3LYP	CCSD (T)	B3LYP	CCSD (T)
HCO_4_^−^ + SO_2_ → HCO_3_^−^ + SO_3_	−28.2	−28.9		
HCO_4_^−^ + SO_2_ → HCO_3_ + SO_3_^−^	−1.7	5.9		
HCO_4_^−^ + SO_2_ → HSO_4_^−^ + CO_2_	−77.5	−81.0		
HCO_4_^−^ + SO_2_ → MIN1	−12.9	−12.7		
MIN1 → MIN2	−2.2	−3.0	−0.7	−0.9
MIN2 → MIN3	−65.8	−72.6	5.0	5.7
MIN3 → MIN4	−1.2	−0.7	4.8	5.3
MIN4 → HSO_4_^−^ + CO_2_	4.7	8.1		
MIN3 → HCO_3_^−^ + SO_3_	52.8	59.4		

## Data Availability

Not applicable.

## Supplementary Materials

The following supporting information can be downloaded at: https://www.mdpi.com/article/10.3390/molecules28010132/s1, Figure S1: Ion–molecule reaction of isolated H^13^CO_4_^−^ ions with SO_2_. Figure S2: Complete geometrical parameters of minima and saddle points relative to the HCO_4_^−^ + SO_2_ PES; Figure S3: Details of MIN1 and MIN2.
