# Supplementary material for: The Peroxymonocarbonate Anion HCO4− as an Effective Oxidant in the Gas Phase: A Mass Spectrometric and Theoretical Study on the Reaction with SO2
Source: Molecules. 2022 Dec 23;28(1):132. doi: 10.3390/molecules28010132 (PMC9822475; doi:10.3390/molecules28010132)
Supplement: Supplementary file 1 [file molecules-28-00132-s001.zip › molecules-2087680-supplementary.pdf]

The Peroxymonocarbonate Anion HCO_4^- as an Effective Oxi-dant in the Gas Phase: A Mass Spectrometric and Theoretical Study on the Reaction with SO_2

Chiara Salvitti [1], Federico Pepi [1], Anna Troiani [1,*], Marzio Rosi [2] and Giulia de Petris [1,*]

[1] Dipartimento di Chimica e Tecnologie del Farmaco, "Sapienza" University of Rome, P.le Aldo Moro 5, 00185 Rome, Italy; chiara.salvitti@uniroma1.it (C.S.); federico.pepi@uniroma1.it (F.P.)

[2] Dipartimento di Ingegneria Civile ed Ambientale, University of Perugia, Via Duranti 93, 06125 Perugia, Italy; marzio.rosi@unipg.it

* Correspondence: anna.troiani@uniroma1.it (A.T.); giulia.depetris@uniroma1.it (G.d.P.)

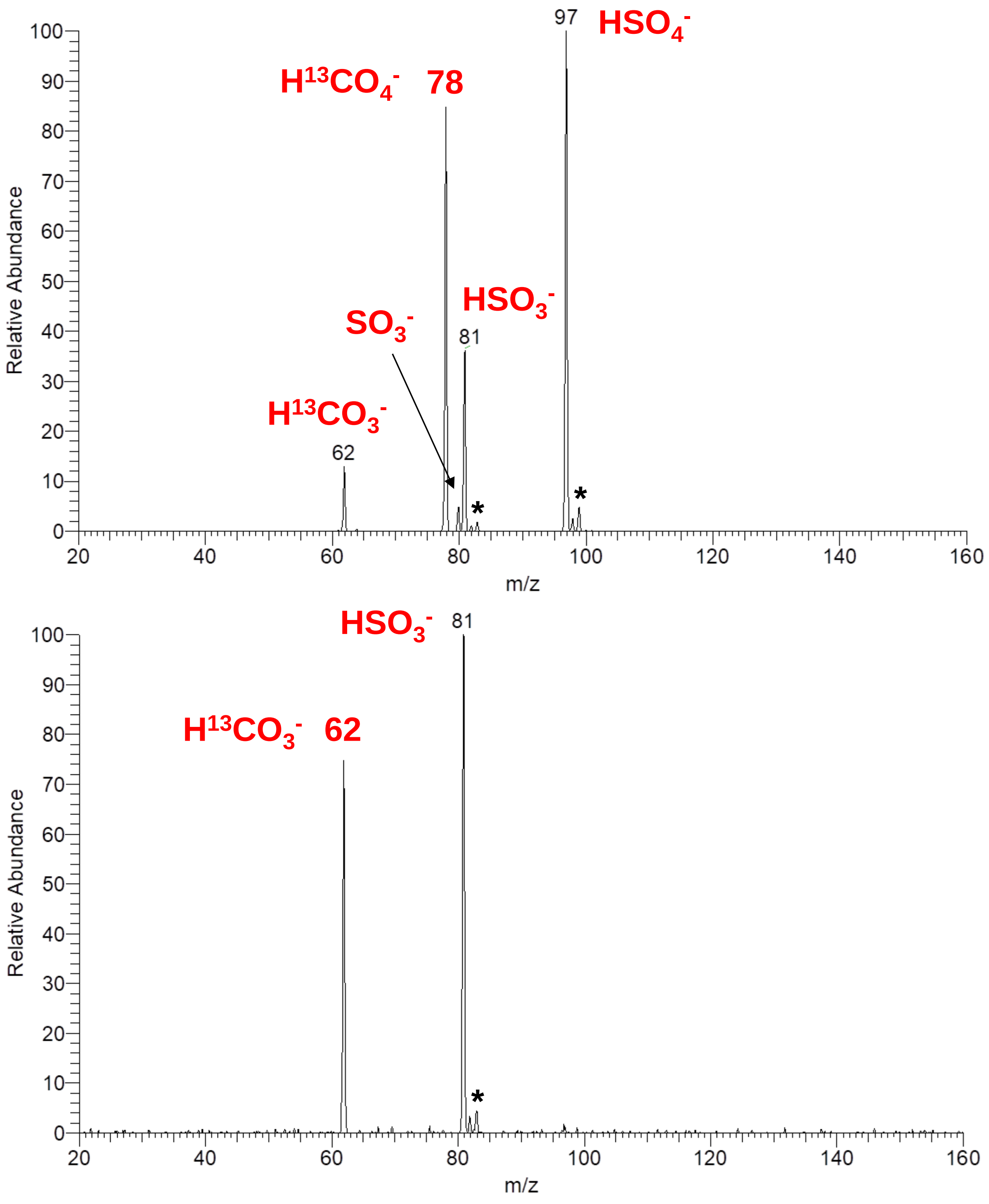

Fig. S1. ITMS spectrum showing (A) the ion-molecule reaction of isolated $H^{13}CO_4^-$ ions (*m/z* 78) with SO_2, reaction time = 200 ms, *P* SO_2 = $1.3x10^{-7}$ Torr. Observed products: HSO_4^- at *m/z* 97, $HSO_3^{\cdot -}$ at *m/z* 81, $H^{13}CO_3^-$ at *m/z* 62; the signals denoted with * correspond to $H^{34}SO_3^-$ (*m/z* = 83) and to $H^{34}SO_4^-$ (*m/z* = 99); (B) the ion-molecule reaction of $H^{13}CO_3^-$ ions (*m/z* 62) isolated from the sequence 78 → 62 with SO_2 (reaction time = 200 ms, *P* SO_2 = $1.3x10^{-7}$ Torr), showing that HSO_3^- at *m/z* 81 is formed from a consecutive reaction. The signal denoted with * at *m/z* 83 corresponds to $H^{34}SO_3^-$.

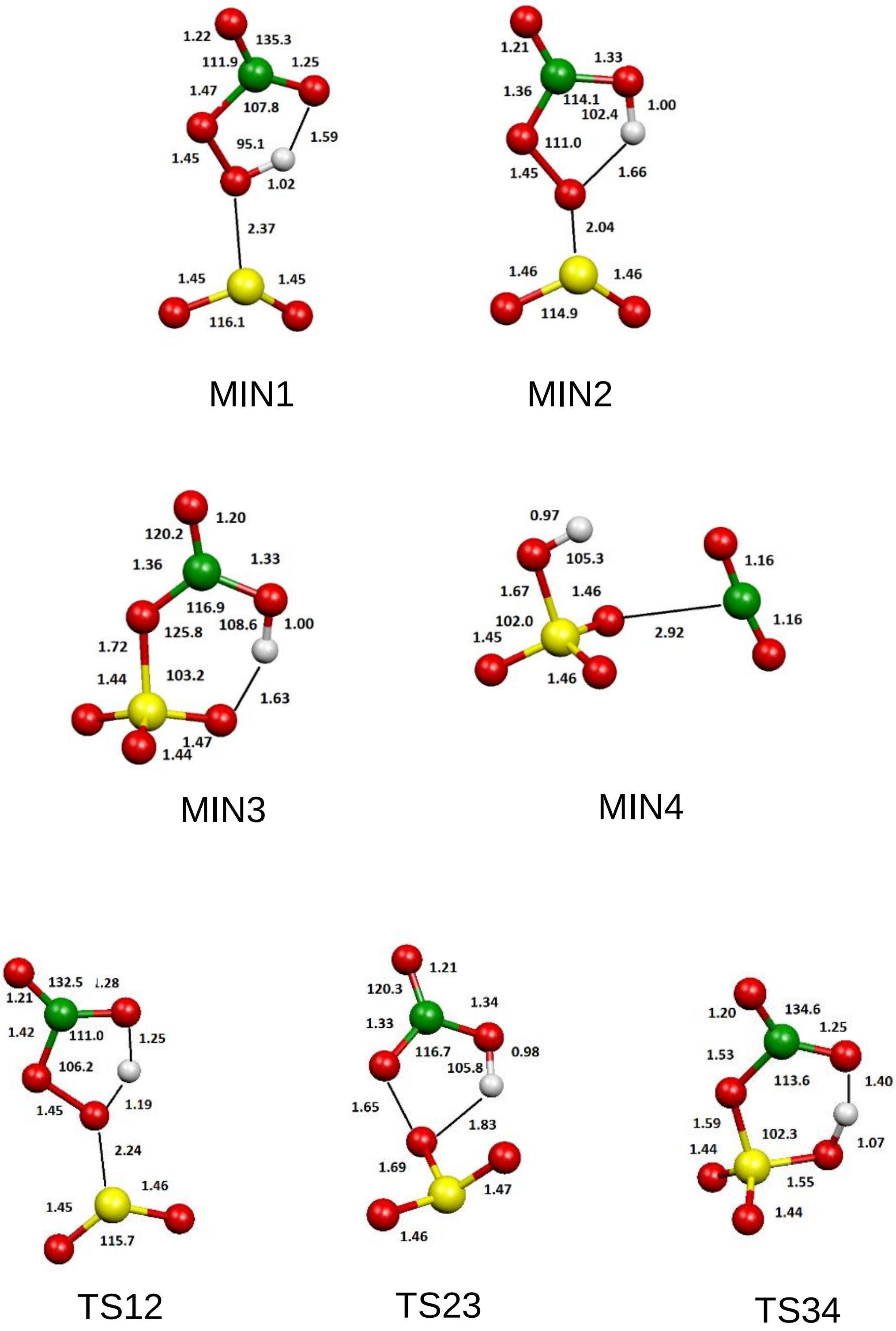

Fig. S2. Complete geometrical parameters of minima and saddle points relative to the HCO_4^- + SO_2 potential energy surface optimized at the B3LYP/aug-cc-pV(T+d)Z level of theory. Bond lengths in Å, angles in degrees.

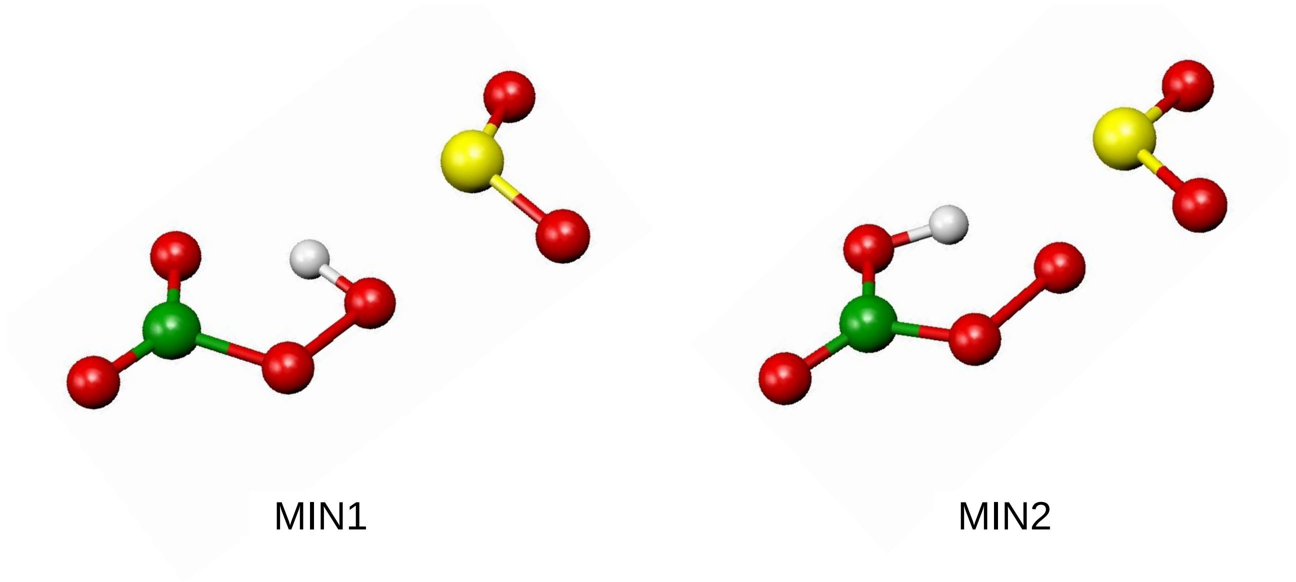

Fig. S3. Interaction between SO_2 and HCO_4^- MIN1 and MIN2 highlighting their bent geometry. In MIN1 the angle between the peroxyl O-S bond vector and the SO_2 plane is around 100°, with the dihedral OSOO angle equal to 102.1°. In MIN2 the angle between peroxyl O-S bond vector and the SO_2 plane is 100°, with the OSOO angle equal to 100.1°.
